# Disentangling the multigenerational transmissions of socioeconomic disadvantages and mental health problems by gender and across lineages: Findings from the Stockholm Birth Cohort Multigenerational Study

**DOI:** 10.1192/j.eurpsy.2023.223

**Published:** 2023-07-19

**Authors:** B. Li, Y. B. Almquist, C. Liu, L. Berg

**Affiliations:** Department of Public Health Sciences, Stockholm University, Stockholm, Sweden

## Abstract

**Introduction:**

There is a paucity of research examining the patterning of socioeconomic disadvantages and mental health problems across multiple generations. The significance of research on multigenerational processes is based on a concern with if and how (dis)advantages are generated and sustained across generations, and how socioeconomic, mental health, and gender inequalities evolve over a longer period of time.

**Objectives:**

The current study therefore aimed to investigate the interconnected transmissions of socioeconomic disadvantages and mental health problems from grandparents to grandchildren through the parents, as well as the extent to which these transmissions differ according to lineage (i.e., through matrilineal/patrilineal descent) and grandchild gender.

**Methods:**

Drawing on the Stockholm Birth Cohort Multigenerational Study, the sample included 21,416 unique lineages by grandchild gender centered around cohort members born in 1953 (parental generation) as well as their children (grandchild generation) and their parents (grandparental generation). Based on local and national register data, socioeconomic disadvantages were operationalized as low income, and mental health problems as psychiatric disorders. A series of path models based on structural equation modelling were applied to estimate the associations between low income and psychiatric disorders across generations and for each lineage-G2 gender combination.

**Results:**

We found a multigenerational transmission of low income through the patriline to grandchildren. Psychiatric disorders were transmitted through both the patriline and matriline, but only to grandsons. The patriline-grandson transmission of psychiatric disorders was partially operated via low income of the fathers. Furthermore, grandparents’ psychiatric disorders influenced their children’s and grandchildren’s income.

**Conclusions:**

We conclude that there is evidence of transmissions of socioeconomic disadvantages and mental health problems across three generations, although these transmissions differ by lineage and grandchild gender. Our findings further highlight that grandparents’ mental health problems could cast a long shadow on their children’s and grandchildren’s socioeconomic outcomes, and that socioeconomic disadvantages in the intermediate generation may play an important role for the multigenerational transmission of mental health problems.

**Disclosure of Interest:**

None Declared

